# Nerve hyperplasia: a unique feature of ketamine cystitis

**DOI:** 10.1186/2051-5960-1-64

**Published:** 2013-10-08

**Authors:** Simon C Baker, Jens Stahlschmidt, Jon Oxley, Jennifer Hinley, Ian Eardley, Fiona Marsh, David Gillatt, Simon Fulford, Jennifer Southgate

**Affiliations:** 1 Jack Birch Unit of Molecular Carcinogenesis, Department of Biology, University of York, Heslington, York YO10 5DD, UK; 2 Departments of Pathology and Urology, St James’s University Hospital, Leeds LS9 7TF, UK; 3 Departments of Pathology and Urology, Southmead Hospital, Bristol BS10 5NB, UK; 4 Urology Department, James Cook University Hospital, Middlesbrough TS4 3BW, UK

**Keywords:** Urothelium, Nerve, p75 nerve growth factor receptor, Neurofilament protein, Bladder pain, Ketamine, Cystitis

## Abstract

**Background:**

There is an emerging association between ketamine abuse and the development of urological symptoms including dysuria, frequency and urgency, which have a neurological component. In addition, extreme cases are associated with severe unresolving bladder pain in conjunction with a thickened, contracted bladder and an ulcerated/absent urothelium. Here we report on unusual neuropathological features seen by immunohistology in ketamine cystitis.

**Results:**

In all cases, the lamina propria was replete with fine neurofilament protein (NFP^+^) nerve fibres and in most patients (20/21), there was prominent peripheral nerve fascicle hyperplasia that showed particular resemblance to Morton’s neuroma. The nerve fascicles, which were positive for NFP, S100 and the p75 low-affinity nerve growth factor receptor (NGFR), were generally associated with a well-developed and in places, prominent, epithelial membrane antigen^+^/NGFR^+^ perineurium. This peripheral nerve fascicle hyperplasia is likely to account for the extreme pain experienced by ketamine cystitis patients. Urothelial damage was a notable feature of all ketamine cystitis specimens and where urothelium remained, increased NGFR expression was observed, with expansion from a basal-restricted normal pattern of expression into the suprabasal urothelium.

**Conclusions:**

The histological findings were distinguishing features of ketamine cystitis and were not present in other painful bladder conditions. Ketamine cystitis afflicts predominantly young patients, with unknown long-term consequences, and requires a strategy to control severe bladder pain in order to remove a dependency on the causative agent. Our study indicates that the development of pain in ketamine cystitis is mediated through a specific neurogenic mechanism that may also implicate the urothelium.

## Background

Ketamine has been used recreationally since the 1970s for its dissociative and hallucinogenic effects. Since it was first reported in 2007 [1], there has been an emerging association between ketamine abuse and the development of severe uro/neurological symptoms including dysuria, frequency and urgency in association with a thickened, contracted bladder (reviewed [2]). Anecdotally, the development of severe bladder pain may impel continued or even increased ketamine usage due to the anaesthetic relief it provides [3]. Ketamine’s illicit status makes this a difficult patient group to study, and so the full extent and incidence of the problem within the population is unknown, and poor documentation of ketamine usage with respect to the development of functional/structural bladder changes has hindered the causal and staged mapping of the pathogenic pathway. Reports of urological symptoms in a minority of patients prescribed ketamine for chronic pain suggests that some individuals may be highly susceptible [4,5].

Histological findings reported to-date for ketamine cystitis include an ulcerated urothelium, neovascularisation, petechial haemorrhages, chronic inflammation/granulation, lymphocytic infiltration, querciphylloid smooth muscle cells (containing peripheral vacuoles) and occasional eosinophilia [1,6-9]. In the absence of an honest case history, ketamine cystitis may be mistaken histologically for urothelial carcinoma in-situ (CIS) due to a disordered morphology and enlarged nuclei, although the application of histopathological markers such as cytokeratin 20 and p53 can differentiate [7].

As an emergent condition where there is a suspected causal agent, it is instructive to compare the pathological features of ketamine cystitis to other benign bladder syndromes. Here we have extended the histopathological study of ketamine cystitis to include further specimens and markers, and compared to a cohort of benign bladder specimens reported elsewhere [10]. This included interstitial cystitis (IC), a chronic and often debilitating inflammatory disorder of the urinary bladder characterised by urinary urgency, frequency and bladder pain, in the absence of infection. As controls, we included non-diseased tissue taken during radical prostatectomy (RP), non-inflammatory dysfunctional conditions of urge urinary incontinence secondary to idiopathic detrusor overactivity (IDO) and stress urinary incontinence (SUI) associated with urodynamic stress incontinence [10].

## Methods

### Tissues

All tissue was collected with NHS Research Ethics Committee approval and either with informed patient consent or was used anonymously. Tissue samples were obtained as cold cut biopsies or cystectomy specimens from patients with clinically-diagnosed ketamine cystitis. Some of the ketamine cystitis specimens have been described previously [7], while others were obtained from James Cook University Hospital.

A control group of bladder biopsies with no history of bladder atypia or malignancy (taken during radical prostatectomies, RP) was included. The series of IC, IDO and SUI specimens has been described previously [10]. Briefly, the non-trigone cold-cut biopsies were obtained from patients diagnosed with IC, urge urinary incontinence secondary to IDO, or SUI secondary to urodynamic stress incontinence, according to published specifications [11,12].

Although there was no statistically significant difference in the mean (range) age for IC, OAB and GSI, at 51 (25–67), 47 (27–71) and 52 (38–80) years, respectively; the ketamine cystitis group was much younger at 26 (19–36) and the RP group older at 71 (61–88).

### Immunohistochemistry

Immunoperoxidase labelling was performed on dewaxed, formalin-fixed 5 μm tissue sections using the antibodies and antigen retrieval methods detailed in Table 1. Blocking steps to neutralise endogeneous peroxidase and avidin-binding activities were included. Antigen retrieval for the antibodies raised against epithelial membrane antigen (EMA), neurofilament protein (NFP) and S100 was performed using “High Retrieval” of 20 min in high pH solution at 97°C (Dako) and labelling was performed with an AutostainerLink 48 (Dako).

**Table 1:** Antibodies used for immunoperoxidase labelling of human bladder biopsies

**Antigen**	**Clone/Catalogue number**	**Species**	**Supplier**	**Concentration(or dilution factor)**	**Antigen retrieval**	**Tyramide amplification**
EMA	E29	Mouse	Dako	1:2000	High retrieval	No
NGFR	7 F10	Mouse	Novocastra	1:100	Citric acid pH6	Yes
NFP	2 F11	Mouse	Dako	1:3000	High retrieval	No
S100	Z0311	Rabbit	Dako	1:6000	High retrieval	No
SMA	1A4	Mouse	Sigma	1:4,000	Trypsin digestion	No

For the p75 low-affinity nerve growth factor receptor (NGFR) antibody labelling, heat mediated antigen retrieval was performed by boiling for 10 min in 10 mM citric acid buffer (pH 6). The sensitivity of detection of NGFR immunolabelling was increased using a tyramide-based amplification system according to the supplier’s instructions (Dako, UK).

For smooth muscle actin (SMA) labelling, trypsin digestion (0.1%, w/v, Sigma) was performed for 10 min at 37°C in 0.1% (w/v) CaCl_2_ (pH 7.8). After overnight incubation in primary antibody at 4°C, slides were washed, incubated in biotinylated secondary antibodies and a streptavidin-biotin horseradish peroxidase complex (Dako Cytomation) and visualised using a diaminobenzidine substrate reaction (Sigma-Aldrich).

All sections were counterstained with haematoxylin, dehydrated and mounted in DPX (CellPath). Positive and negative specificity controls were included in all experiments.

## Results

### Neurogenesis in ketamine cystitis stroma

**Figure 1:**
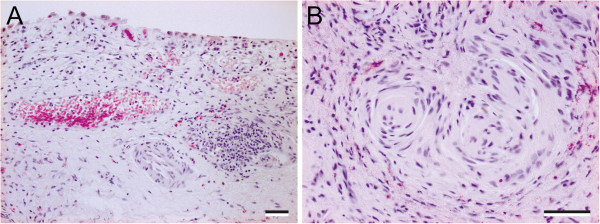
**Representative images from haematoxylin and eosin stained sections of ketamine cystitis tissue. (A)** Typical ketamine-induced changes with increased sub-epithelial capilliarisation and oedema of the lamina propria. In this sample there is early lymphoid aggregate formation which is not uncommon. The overlying urothelium is reduced to a patchy single layer of epithelial cells showing atypical cytological changes. **(B)** Lamina propria with prominent nerve fascicles and conspicuous perineurium; some eosinophils in the background. Scale bar represents 100 μm.

Urothelial damage was a notable feature of all ketamine cystitis specimens, with most showing partial loss of superficial cells and many containing focal full-thickness urothelial loss (Figure 1A). Haematoxylin and Eosin staining also revealed prominent nerve fascicles in the lamina propria of ketamine cystitis patients (Figure 1B).

**Figure 2:**
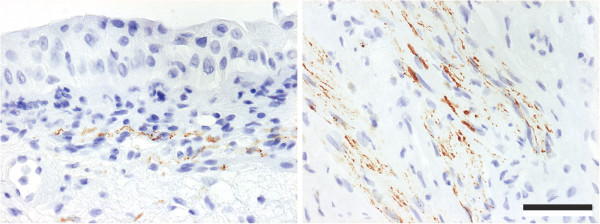
**Representative images of NFP immunohistochemistry in bladder biopsies from ketamine cystitis patients, showing positive fibres in close proximity, but not infiltrating, the urothelium (left) and large clusters of fibres in the lamina propria (right).** Scale bar represents 100 μm.

The stroma of every ketamine cystitis patient tested was replete with fine NFP^+^ nerve fibres (Table 2). In some cases, the nerve fibres were observed in close proximity to the urothelium, although no fibres could be detected crossing the basement membrane into the epithelial compartment (Figure 2). Patchy/weak NFP^+^ labelling was occasionally detected in the stroma of IDO, SUI and IC samples (Table 2).

**Table 2:** Table summarising graded results of immunolabelling

**Histopathlogical feature**	**IDO**	**SUI**	**IC**	**Ketamine cystitis**
Retention of basal and intermediate urothelial cells in some areas	5/6	3/4	11/11	16/21
Intense supra-basal expansion of NGFR	1/5	1/3	0/11	10/16
NFP^+^ in the lamina propria	1/6	1/4	1/11	21/21
Bladder wall neuroma	0/6	0/4	0/11	20/21

**Figure 3:**
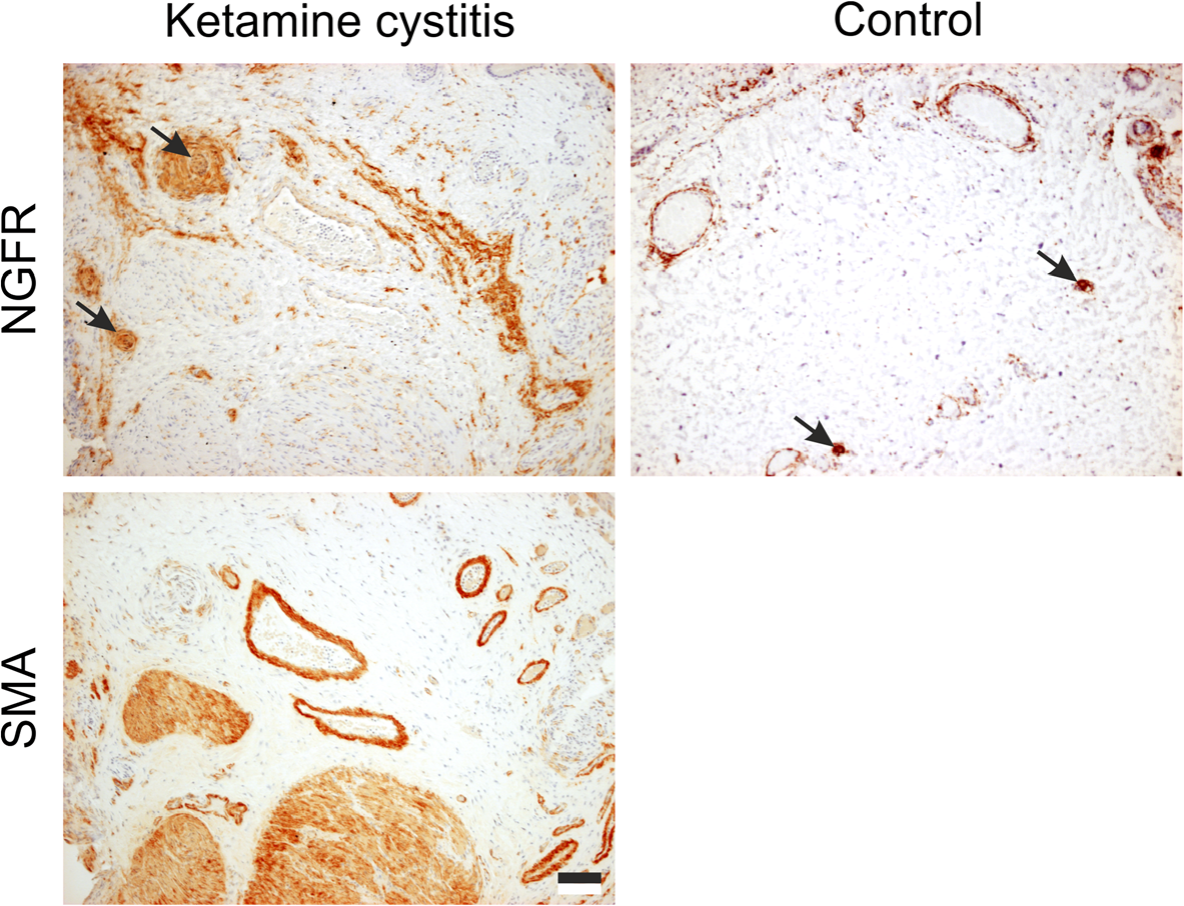
**Representative images of NGFR and SMA immunohistochemistry showing the stromal NGFR**^**+ **^**cells in ketamine cystitis tissue and a serial section demonstrating that NGFR**^**+ **^**cells are SMA**^**-**^**.** SMA^+^ labelling of vascular walls also highlights the appearance of prominent blood vessels in the bladder wall of ketamine cystitis biopsies. NGFR^+^ peripheral nerve fascicles are arrowed in ketamine cystitis and “control” non-diseased bladder taken during radical prostatectomy. Scale bar represents 100 μm.

NGFR labelling of RP/IDO/SUI bladder stroma was mostly confined to the blood vessel walls, occasional interstitial cells and nerve fascicles (arrowed, Figure 3). NGFR^+^ labelling was abundant in the stroma of ketamine cystitis tissues with varying intensity which could not be directly associated with local urothelial loss. The NGFR^+^ lamina propria cells observed in ketamine cystitis were similar morphologically to myofibroblasts, but were negative for SMA in serial sections (Figure 3). NGFR^+^ labelling also revealed prominent peripheral nerve fascicle hyperplasia in 20 of 21 ketamine cystitis biopsies (arrowed, Figure 3), but not in the other bladder pathologies studied (summarised Table 2).

**Figure 4:**
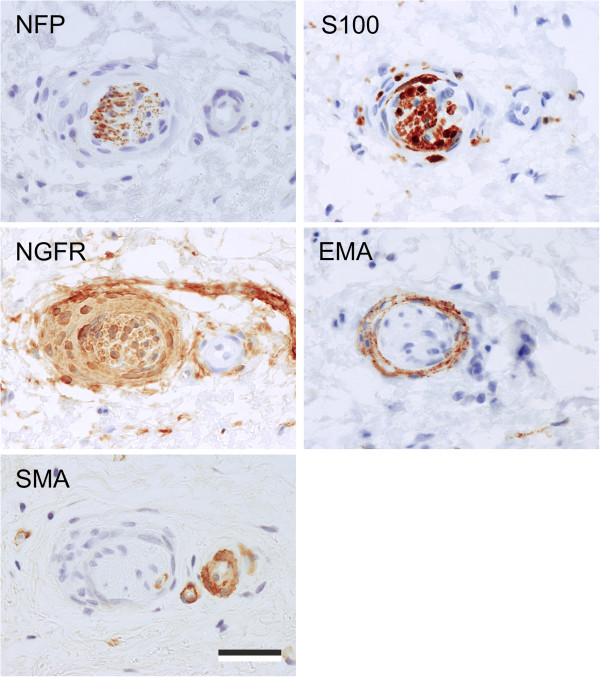
**Proximal sections showing an example of the NFP**^**+**^**, NGFR**^**+ **^**and S100**^**+ **^**labelling patterns in peripheral nerve fascicle identified in ketamine cystitis tissue and observed in nearly all ketamine cystitis samples.** EMA showed a perineural labelling pattern. The prominent perineural spindle cell cuff was also NGFR^+^ and exhibited scanty SMA^+^ restricted to occasional cells. Note, the adjacent blood vessel, which showed SMA labelling of the mural smooth muscle cells, but unlike the nerve fascicle was NFP^-^, NGFR^-^ and S100^-^. Scale bar represents 100 μm.

Further investigation revealed that the NFP^+^/S100^+^/NGFR^+^ nerve fascicles were generally associated with a well-developed, in places prominent EMA^+^/NGFR^+^ perineurium (Figure 4). No Schwannian vaculolar change or inflammation was noted. The nerve fascicles were usually identified in the deeper portion of the lamina propria or within the detrusor muscle, but occasionally also in the superficial lamina propria (Figure 3).

In places the nerve fascicle-like structures displayed features reminiscent of the regenerative interstitial changes in peripheral nerve fascicles seen in neuromas.

### Urothelial NGFR expansion

**Figure 5:**
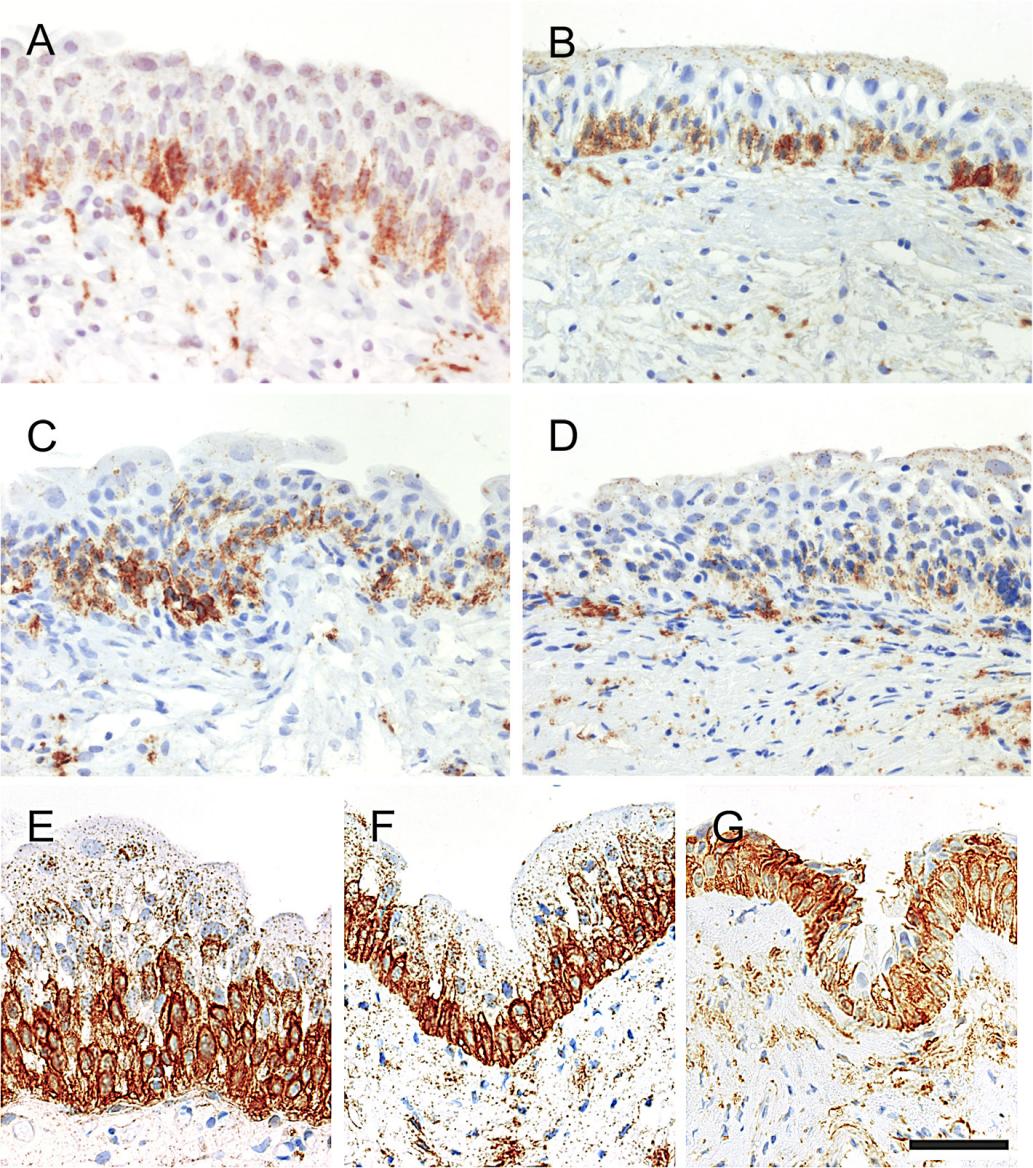
**Representative images of NGFR immunohistochemistry showing urothelial localisation in a range of bladder conditions including “A” non**-**diseased bladder taken during radical prostatectomy, “B” idiopathic detrusor overactivity and “C” stress urinary incontinence and “D” interstitial cystitis.** Supra-basal expansion of NGFR labelling was only occasionally observed in idiopathic detrusor overactivity and stress urinary incontinence (see Table 2). In ketamine cystitis biopsies (“**E**”, “**F**” & “**G**”), supra-basal expansion of the intense NGFR labelling was observed in 10 of the 16 patients who retained intermediate urothelial cells. Scale bar in panel “G” represents 100 μm.

NGFR expression was predominantly basally-restricted, but patchy, in RP, IDO and SUI samples (Figure 5A, B and C). In 8/11 IC samples, the normal intense basal pattern was replaced by a weak punctuate labelling throughout the urothelium (Figure 4D).

In the ketamine cystitis patient group, 16 retained urothelium including intermediate cells and in 10 of these samples, expansion of intense basal NGFR expression into the intermediate compartment was observed (Figure 5E-G). In the 6 remaining ketamine cystitis samples the NGFR expression was comparable to RP/IDO/SUI tissues. Where supra-basal expansion of NGFR^+^ labelling was observed it frequently included all but the most superficial layer (example in Figure 5F&G). This expansion was only occasionally observed in the other benign bladder pathology biopsies tested (Table 2).

## Discussion

Ketamine cystitis is a growing global problem afflicting predominantly young patients and exposing them to significant risk of bladder damage with unknown long-term consequences. To-date, there has been little research into the pathology of ketamine cystitis and as a result, the mechanism(s) of the bladder pain and damage remain unknown. This histological study observed expansion of the basal NGFR^+^ labelling, stromal nerve hyperplasia and the occurrence of superficial neuroma-like lesions which likely contribute to the extreme bladder pain experienced by ketamine cystitis patients.

The discovery of numerous fine NFP^+^ nerve fibres throughout the stroma of ketamine cystitis tissues is unusual and to our knowledge has not been previously described. The presence of nerve hyperplasia in ketamine cystitis tissue in conjunction with urothelial damage leading to stromal urine exposure may help to account for the extreme pain experienced by ketamine cystitis patients. Understanding the mechanism of pain in ketamine cystitis is critical to developing effective new treatment strategies since at present, many ketamine users self-manage their pain with increased ketamine use. The current lack of effective clinical pain management for these patients is a key obstacle to cessation of use [2]. In neuropathic bladders, there have been reports of nerve hyperplasia invading the urothelium [13]; however, no NFP^+^ fibres were observed within the urothelium in this study. In the small group of IC patients studied here only a single sample contained visible NFP^+^ fibres in the lamina propria. That this NFP^+^ IC patient may have been an undisclosed ketamine user cannot be ruled out; however, the potential utility of NFP as a biomarker for an IC subgroup with similarities of pathogenesis to ketamine cystitis warrants further investigation. Based on current knowledge, the discovery of NFP^+^ fibres in the bladder stroma may be a useful, if not unequivocal, clinical biomarker of ketamine cystitis in patients who have non-bacterial cystitis, but do not provide a history of drug use.

A further novel, and apparently unique, feature of ketamine cystitis reported here is the appearance of large peripheral nerve fascicles in the lamina propria, with a predominant Schwannian and perineural component, and some resemblance to a Morton’s neuroma. These lesions appear to arise as a hyperplastic/reactive response and may be consequential to interstitial regeneration following ketamine damage. At present, it is unclear how these changes relate to the degree of pain experienced in these patients; however, they appeared in nearly all (20/21) urology-referred ketamine cystitis patients in this study and were not seen in the other bladder pathologies studied as controls.

The cause of peripheral nerve fascicle hyperplasia in ketamine cystitis tissues remains unknown; however, chronic ketamine users (of at least 4 times/week) have on average twice the serum concentration of brain-derived neurotrophic factor (BDNF) when compared with a control group [14]. Further study of ketamine cystitis will need to address whether the cause of nerve fascicle hyperplasia is the direct action of ketamine and/or its metabolites; or alternatively, whether circulating BDNF could be the causative agent.

The role of NGFR in the urothelium remains an interesting unknown; however, in RP/IDO/SUI tissues it is most commonly confined to basal urothelial cells. The expansion of NGFR^+^ might be indicative of a general dedifferentiation of the tissues; however, no change was noted for other basal markers (eg CK5) and there was no disruption of differentiation markers such as uroplakin 3a (data not shown). Previous studies have reported increased Ki67 indices in ketamine cystitis urothelium [7] and interpreted with the supra-basal NGFR^+^ expansion reported here, this might suggest changes in the epithelium towards a regenerative wound-healing phenotype. This concept is consistent with the widespread urothelial damage observed in ketamine cystitis and retention of uroplakin labelling suggests the urothelium retains a functional barrier in areas where it remains full-thickness. During cystoscopy of one patient the urothelium was observed desquamating from the basement membrane as large sheets, which was consistent with finding histologically that areas of intact full-thickness urothelium were directly adjacent to areas of absent urothelium. Whether the mechanism of urothelial loss relates to direct toxicity of ketamine or the action of a metabolite requires further study.

Whilst ketamine was originally described as a NMDA receptor antagonist, this is a gross oversimplification of its binding promiscuity, which includes activity against β-adrenergic, sigma, and muscarinic receptors [15]. Recent interest in using ketamine as a rapid-onset anti-depressant and pressure to drive derivatives to market quickly (reviewed [16]), make understanding the mechanism of ketamine cystitis an urgent clinical problem to avoid side-effects in future therapeutics.

## Conclusions

The results of this study indicate that the severe bladder pain associated with chronic exposure to ketamine derives from the combination of a compromised urinary barrier and widespread nerve hyperplasia in the bladder wall, including the development of a novel neuroma with similarities to Morton’s neuroma. This study has also shown that the neurogenic changes seen in ketamine cystitis extend to the damaged urothelium, where increased expression of NGFR makes an unknown contribution to the pathology. Ketamine cystitis is a debilitating pathology with long-term consequences for affected young persons and urgently requires further research to develop effective treatments.

## Abbreviations

EMA: Epithelial membrane antigen; IC: Interstitial cystitis; IDO: Idiopathic detrusor overactivity; NFP: Neurofilament protein; NGFR: p75 low-affinity nerve growth factor receptor; RP: Bladder sample taken during radical prostatectomy; SMA: Smooth muscle actin; SUI: Stress urinary incontinence.

## Competing interests

The authors declare that they have no competing interests.

## Authors’ contributions

SCB, JSt and JH carried out experiments, SCB, JSt and JSo conceived experiments and analysed data. SF, IE, FM, JO and DG supported the acquisition of data. All authors were involved in writing the paper and had final approval of the submitted and published versions.
